# Experiences and perceptions of people with a severe mental illness and health care professionals of a one-year group-based lifestyle programme (SMILE)

**DOI:** 10.1371/journal.pone.0271990

**Published:** 2022-08-04

**Authors:** Florine Walburg, Johanna Willemina de Joode, Hella Brandt, Maurits van Tulder, Marcel Adriaanse, Berno van Meijel

**Affiliations:** 1 Department Health Sciences, Amsterdam Public Health Research Institute, Vrije Universiteit Amsterdam, Amsterdam, The Netherlands; 2 Department Human Movement Sciences, Faculty of Behavioural and Movement Sciences, Vrije Universiteit Amsterdam, Amsterdam, The Netherlands; 3 Department of Health, Sports & Welfare, Research Group Mental Health Nursing, Inholland University of Applied Sciences, Amsterdam, The Netherlands; 4 Department of Psychiatry, Amsterdam Public Health Research Institute, Amsterdam UMC (VUmc), Amsterdam, The Netherlands; 5 Parnassia Psychiatric Institute, Parnassia Academy, The Hague, The Netherlands; University of Toronto, CANADA

## Abstract

**Objective:**

This was to elucidate the experiences and perceptions of people with severe mental illness (SMI) and their health care professionals with the SMILE (Severe Mental Illness Lifestyle Evaluation) group-based lifestyle intervention. SMILE focuses primarily on promoting healthy diet, physical activity and weight loss.

**Method:**

A qualitative study with semi-structured interviews was conducted using purposive sampling. Interviews were conducted with 15 clients and 13 health care professionals (HCPs). Data were analysed according to a thematic analysis.

**Results:**

Four overall themes were identified: interest in a lifestyle programme; group-based setting; changes in lifestyle behaviour; and preconditions for changing health behaviour. The results showed that clients valued the programme and were interested in the subject of lifestyle. The group-based setting was seen as a positive and important aspect of the intervention. Making lifestyle changes was acknowledged as difficult, especially in combination with the presence of psychiatric symptoms. Clients acquired an improved awareness of different aspects related to lifestyle behaviour. Irrespective of weight loss achieved, clients found their efforts successful with relatively ‘small’ changes. Some needed more support during the intervention than others. The practical activities in group sessions were regarded as most useful. HCPs were enthusiastic about the programme and their interactions with lifestyle improvements.

**Conclusions:**

The results of this study shed light on different aspects that were considered important when delivering a lifestyle intervention to people with SMI. We recommend considering these aspects when implementing a lifestyle intervention in a mental health care setting for clients with SMI.

## Introduction

People with a severe mental illness (SMI) have an increased risk of premature mortality, with cardiovascular disease being one of its primary causes [1, 2]. Factors associated with this increased risk include, amongst others, unhealthy lifestyle behaviours and adverse effects of treatment, in particular use of antipsychotic medications which cause weight gain and metabolic abnormalities [3–5]. People with SMI tend to be less physically active than the general population, be more sedentary, have a lower consumption of fruit and fibre and a higher consumption of sugars and saturated fat [3, 5–7].

Improvements in nutrition and physical activity may have significant benefits for the physical and mental health of people with SMI [8]. Furthermore, a healthy lifestyle can contribute to feelings of empowerment, health-related self-efficacy, feelings of greater autonomy, and improved social integration and quality of life [3, 8–10].

Sustainable changes in lifestyle behaviour are difficult to achieve for most people. People with SMI face additional challenges in achieving these due to the symptoms of the psychiatric disorder, and their consequences for personal and social functioning [8, 11]. Sufficient time and support, with a focus on incremental changes in lifestyle behaviour, are needed for achieving long-term effects [11–13].

Many lifestyle interventions for people with SMI focus on weight loss and cardiovascular risk reduction. These interventions are mostly delivered in highly controlled study settings [14]. Consequently, aspects related to real-world settings in which clients function, as well as the experiences of clients of their participation in such lifestyle improvement programmes, have been little researched. For an effective lifestyle programme, the principle of tailoring the delivery of the intervention to the specific needs and preferences of individuals is essential, including consideration of influential factors from the real-world settings of clients.

Further research is needed to better understand what is important for people with SMI when engaging in lifestyle intervention programmes, in order to identify important factors to consider when tailoring lifestyle interventions to their specific needs.

We studied an extensive lifestyle programme, with a focus on a healthy diet, physical activity and weight loss, called the Severe Mental Illness Lifestyle Evaluation (SMILE) intervention. The intervention was performed during ambulatory care for people with SMI, the (cost-)effectiveness of the programme also being studied [15]. In parallel, a process evaluation was performed [16], describing the implementation process of the intervention, with an elaboration of barriers and facilitators for its effective implementation. The current study is a sub-study of the process evaluation performed with the aim of elucidating the experiences and perceptions of people with SMI and of their health care professionals (HCPs) with the SMILE intervention. Though clients’ perspectives are central in this study, we also interviewed the HCPs delivering the SMILE intervention in order to explore their experiences in parallel with those of their clients. The findings may shed more light on how to improve tailoring of lifestyle interventions to people with SMI.

## Method

### Study design

A qualitative research design with semi-structured interviews was used to study the experiences and perceptions of patients with SMI and of their health care providers with the SMILE intervention. This study was performed alongside a pragmatic cluster randomized controlled trial (RCT) evaluating the cost-effectiveness of the SMILE intervention in Dutch ambulatory mental health care, in combination with a process evaluation studying the implementation process of the intervention [16]. Eleven ambulatory FACT (Flexible Assertive Community Treatment) teams participated in the intervention arm of the SMILE trial which was based on the STRIDE study [17, 18]. For a detailed description of the SMILE study design, see Walburg et al., 2019 [15].

### SMILE intervention

The SMILE intervention is a one-year intervention, with weekly two-hour group sessions during the first six months (initial phase) and subsequent monthly group sessions, combined with individual telephone support during the last six months (maintenance phase). It mainly focused on establishing a healthy diet, promoting moderate physical activity, and losing weight, but additionally targeted other aspects of lifestyle, such as sleep, stress, negative thinking and social support. An overview of the content of the SMILE intervention group sessions is presented in the S1 File. Group sessions, varying in size from 7 to 16 clients, were delivered by two trained mental HCPs. Their format was as follows: sessions started with a check-in, where successes and challenges experienced during the period between the sessions were discussed; this was followed by discussing one or two topics scheduled for that week; finally, participants formulated and discussed their personal goals for the upcoming week or month; there was also a 20–30 minute workout, including walking with the group or performing indoor exercises. All FACT team clients were eligible to participate in the intervention if they had a BMI of 27 or higher and were 18 years or older.

### Procedures

We interviewed clients with SMI who participated in the SMILE intervention and HCPs who delivered the intervention. We included the HCPs’ perspective to improve the credibility of the findings, though the clients’ perspectives were our primary focus. We included at least one client and one HCP from each of the 11 FACT teams.

To achieve sufficient variation in the sample of clients, with a wide range of different perspectives, we used a purposive sampling strategy with the following criteria: variation in (1) attendance at group sessions, (2) challenges experienced by the client during the SMILE intervention, (3) weight change after six months, (4) gender and (5) diagnosis. All but one of the clients interviewed completed the full SMILE intervention. We included both women and men with high or low attendance, high and low weight change, and different mental health diagnoses. We also included a client who had dropped out of the study. We included HCPs based on their active involvement in the implementation of the SMILE intervention, creating a sample of all disciplines involved in the trial: mental health nurse, expert-by-experience, social worker, activity worker, and psychologist.

We used different interview guides to conduct semi-structured interviews with clients and HCPs (see S1 File). These guides were based on topic lists, the topics being based on the RE-AIM framework as this study was performed alongside the process evaluation which focuses on the process of implementation of the intervention. The RE-AIM framework assesses five dimensions to enhance the quality, speed, and public health impact of efforts to translate research into practice [19]. These are Reach, Efficacy, Adoption, Implementation, and Maintenance (hence RE-AIM). We sought to elucidate the perceptions and experiences of interviewees with the SMILE intervention along these dimensions to gain insight into the different elements of the delivery of an intervention. These RE-AIM dimensions were incorporated throughout the interviews but were not explicitly used for the analysis of the data in the present study, given our focus on the experiences of clients and professionals. Data were collected and analysed using an iterative process, in which topics were adapted throughout the interview period, and new topics added in subsequent interviews, based on information collected in previous interviews. All interviews took place near the end of the SMILE intervention period. Data collection stopped when no new information was derived from interviews (data saturation) [20]. Interviews were audiotaped, transcribed verbatim and anonymized. A member check was performed to check the credibility of the data: participants were sent a summary of their interview and asked if they recognized the main themes described.

### Data analysis

Data were analysed using thematic analysis [21, 22]. All transcripts of the interviews were coded separately by two researchers (FW and JWdJ). MAXQDA 2018 software was used to facilitate data analysis. The analysis comprised six phases: (1) familiarization with the data by reading and summarizing all transcripts; (2) the generation of initial codes; (3) searching for themes; (4) reviewing whether themes credibly represented the data; (5) defining and naming the themes; and (6) producing the report. Themes derived from the analysis were compared and discussed by both researchers until consensus about the central themes was reached. Overall, there was a strong consistency between both researchers. A third researcher (HB) with extensive experience in the field of qualitative research contributed to the analyses in case of lack of consensus between the two initial researchers.

### Ethics

All participants gave written informed consent in accordance with the Declaration of Helsinki. The study received ethical approval from the Medical Ethical Committee of the VU University Medical Centre in Amsterdam, the Netherlands (NL60315.029.17, registration number 2017.418).

## Results

### Participants

A total of 28 interviews were conducted, 15 with clients and 13 with HCPs. Clients interviewed were between 30 and 61 years old; nine were female and six male. Ages of the HCPs interviewed ranged from 30 to 62 years; ten were female and three male. Details of the characteristics of the HCPs and clients are presented in Tables 1 and 2, respectively. Interviewees could choose whether the interview took place in the mental health care institute or at home. Interview durations ranged from 22 to 53 minutes with clients and 38 to 75 minutes with HCPs. Seven (48%) of the client interviewees and all but one (92%) HCP interviewees responded to the member check, and agreed with the summaries. From the interviews, we identified four main themes and eight subthemes.

**Table 1:** Characteristics of interviewed health care workers.

No.	Gender	Age	Discipline	Total sessions given
HCP1	Male	60–69	Nurse	15
HCP2	Female	40–49	Activity coordinator	20
HCP3	Female	50–59	Nurse	23
HCP4	Female	30–39	Nurse	24
HCP5	Female	50–59	Nurse	30
HCP6	Female	50–59	Nurse	28
HCP7	Female	30–39	Social worker	27
HCP8	Female	30–39	Psychologist	25
HCP9	Female	30–39	Expert-by-experience	29
HCP10	Female	30–39	Nurse	28
HCP11	Male	50–59	Nurse	27
HCP12	Female	50–59	Nurse	27
HCP13	Male	40–49	Nurse	24

**Table 2:** Characteristics of interviewed clients.

No.	Gender	Age	Diagnosis	Weight change after 6 months	Total attendance
C1	Male	50–59	Schizophrenia or other psychotic disorder	Gain	18
C2	Female	40–49	Borderline or other personality disorder	Loss	23
C3	Female	30–39	Borderline or other personality disorder	Loss	23
C4	Male	50–59	Schizophrenia or other psychotic disorder	Loss	14
C5	Male	40–49	Schizophrenia or other psychotic disorder	Gain	18
C6	Female	40–49	Depressive or bipolar disorder	Gain	3
C7	Female	50–59	Depressive or bipolar disorder	Loss	28
C8	Female	30–39	Schizophrenia or other psychotic disorder	Gain	11
C9	Male	40–49	Schizophrenia or other psychotic disorder	Loss	29
C10	Female	40–49	Depressive or bipolar disorder	Equal	23
C11	Female	50–59	Schizophrenia or other psychotic disorder	Loss	20
C12	Female	50–59	Post-traumatic stress disorder	Loss	21
C13	Female	30–39	Post-traumatic stress disorder	Loss	21
C14	Male	50–59	Schizophrenia or other psychotic disorder	Loss	30
C15	Male	60–69	Obsessive compulsive disorder	Loss	29

### Theme 1: Interest in a lifestyle programme

#### Clients valued the programme

Changing lifestyle behaviour and losing weight were subjects of interest for clients. Many had (repeatedly) attempted to improve their lifestyle behaviour and lose weight before joining the SMILE intervention. Generally, clients linked their overweight and their struggles with lifestyle behaviour to aspects of their mental illness, such as symptoms of their disorder and their medication use. For example: one client stated that a healthy lifestyle was important for him because his obsessive-compulsive disorder had a negative impact on his lifestyle behaviour.

*"I think it’s good anyway to live a healthy life*, *to exercise a lot and work on my diet, because my lifestyle is dictated quite a lot by my compulsions" (C15)*.

The consensus was that clients enjoyed participating in the sessions and actively worked on their lifestyle during the SMILE intervention. The sessions were experienced as stimulating and regarded as a positive way to encourage behavioural lifestyle changes. The main motivation for clients to join the intervention was to lose weight and improve their physical health in general. In addition, some clients specifically mentioned the need to gain more knowledge about lifestyle-related issues, such as healthy foods and physical activity. They wanted to feel better and to increase their awareness of how to achieve this. Motivational factors mentioned by a minority of clients were more practical in nature, such as having the time available and the opportunity to join in.

### Theme 2: Group-based setting

#### The group-based setting had many benefits

The group-based setting of the intervention was attractive for clients. Some mentioned it as one of the main motivators for joining the intervention. The group sessions were perceived as pleasant and safe to exchange their experiences and challenges in their efforts for lifestyle changes and to discuss related aspects of their mental health. Clients indicated the importance of peer support from their fellow group members which contributed to the experience of a collective effort to change lifestyle behaviour. This motivated them to attend the sessions.

*"And you really do it together, you know*. *It is a long process that you go through. You share experiences with each other. And I think that’s what is so nice" (C13)*.

Clients recognized themselves in the shared struggles of the other group members with lifestyle changes. The sense of community was enhanced by the fact that they shared common characteristics, such as being overweight and experiencing mental health problems, both central topics of discussion during the sessions. The exemplary behaviour of one client could contribute to the behaviour change of another.

*"I felt like I belonged in the group, because we have all common experiences*, *and I thought it was nice to have people around me with the same experiences" (C8)*.

*"Recognition. And you could also see that someone who, for example*, *experienced binge eating, then went for a walk instead. And then I said to myself: I’ll do that as well next time" (C13)*.

In addition, clients said that the group setting brought them into contact with other people, which was seen as a positive experience. Some clients stated they started seeing other group members outside the group sessions, which led to new friendships.

The positive aspects of the group setting were also recognized by HCPs. They underlined the ‘power of the group’ with its supportive and helping effects on the clients.

*"And I think that is really the strength of a group, that they feel very, very supported by everyone there*. *Because everyone understands exactly what they are talking about, because they’re all willing to show their vulnerable side. Everyone has the same problem. So it feels quite powerful, leading to change, being with people who are in the same boat" (HCP12)*.

#### Clients felt safe with the HCPs and felt they were part of the group

Clients felt positive about the HCPs who delivered the intervention. They noted that HCPs were emotionally and practically involved with their challenging efforts for lifestyle changes and the struggles they went through as clients.

*"The climate during the sessions was very supportive, they really paid attention to you*. *Yes, a lot of attention for every person, you never felt excluded from the group" (C8)*.

Clients appreciated the coaching and the concrete advice HCPs gave them. They also valued the personal stories HCPs shared about their own struggles with lifestyle changes. This stimulated group cohesion: common experiences of both clients and HCPs could be shared, which led to normalization of these experiences and open discussions about possible solutions to manage lifestyle challenges. It was felt that it was important that the gap between the lifestyle behaviour of clients and HCPs not be too great, i.e., HCPs as prototypes of healthy persons versus the clients with overweight and poor lifestyle habits:

*"If there had been two group leaders who were very fanatical about sports and already very health-conscious in terms of nutrition*, *it would have been very counterproductive" (C12)*.

For HCPs, group cohesion was also a recurring subject during the interviews. They were enthusiastic about their interaction with the clients and felt as if they were a part of the group, rather than being ‘group leaders’.

*"I thought it was important that we, the group leaders, also shared our experiences, that we also were confronted with poor lifestyle behaviours that were difficult for us to change*. *People appreciate it when you say: I can’t stay away from that candy jar or something. So, I thought that was also a strength. That you are part of the group" (HCP12)*.

### Theme 3: Changes in lifestyle behaviour

#### Making changes was difficult

Changing lifestyle behaviour and losing weight was acknowledged as difficult by clients, especially in combination with the presence of psychiatric symptoms.

*"For me, it is a little more difficult to lose weight, in particular in periods of depression*. *Then, exercising is a major challenge. And that was also the big stick, that you are with the group and that you can drag each other through. So yes, I found that very pleasant" (C3)*.

During the intervention, clients learned to formulate their personal goals. They observed that it was difficult to formulate attainable goals and stick to them. One said:

*"I found it very difficult at first*, *but that’s because I wanted a faster pace to make greater headway" (C12)*.

HCPs affirmed the complexity for clients of making changes, mentioning as one reason the diversity in levels of cognitive functioning within groups, with some clients having trouble concentrating during sessions. In their view, there needed to be sufficient time for all clients to understand the central topics discussed in each session, and to stimulate them to formulate small concrete attainable goals, leading to experience of success.

*“But, in that case, it is important to realize that you have to set the goals quite low*. *Small goals and not too elaborate. And it is useful, especially with the SMI target group, to specify those very clearly, because they often have a lot of difficulty formulating clear and achievable goals" (HCP7)*.

Furthermore, HCPs noted that when clients’ goals were achieved, even though modest, it was seen as a great success by the clients.

*"And I have to say that it was quite nice*, *because people are also very happy when they have reached that small goal" (HCP13)*.

#### Improved awareness of own lifestyle behaviour and how to change it

The most common change clients experienced was a greater awareness of several aspects related to lifestyle behaviour, such as enhanced understanding and awareness of current food intake, unhealthy foods, the function of emotional eating and other barriers for lifestyle behaviour change. This change that was regarded as an important and helpful development. One client said:

*"And all those things regarding increasing awareness about lifestyle issues*, *that was most beneficial for me" (C12)*.

This increased awareness applied, among other things, to emotional eating, a frequently recurring issue for clients throughout the sessions. Clients commented that they had learned about the triggers and functions of emotional eating, and how to prevent this form of unstructured and excessive food intake. This was seen as a struggle for both clients and HCPs.

*"She explained how it works if you are an occasional ‘emotional eater’*. *You are angry about something and you snack a little bit and then you feel guilty*. *And they have explained so well how it works*. *You can get a setback" (C11*).

#### For clients, weight loss was not the most important outcome

Clients were happy when losing weight but the interviews revealed that clients did not experience this as the most important outcome of the lifestyle intervention. Irrespective of weight loss achieved, clients felt their efforts to be successful even with ‘small’ lifestyle changes in different areas of functioning. One said:

*"I have lost some weight, of course, which is great but that is not the most important thing for me*, *but rather the balanced meals and the small steps with exercise, and that you just become more aware of that" (C3)*.

Clients experienced a wide range of changes throughout the intervention, which they appreciated. Changes related to physical activity and nutrition were: more exercise, less snacking, higher fruit consumption, smaller portion sizes, and consciously choosing healthier food alternatives. Interestingly, clients also mentioned changes that had a positive effect on their daily lives in general. For example, one mentioned a positive effect on her daily routine:

*"I still used to sleep in every day but since the SMILE intervention I am often up in the morning at seven, half past seven*. *And then I’ll have my breakfast earlier. Because before, it was as late as 11 o’clock. And I do that earlier in the morning now. So I did change that. My daily routine has improved, let me put it that way" (C7)*.

Another common topic amongst clients was the improvement in general wellbeing. For example:

*"Through the SMILE-intervention, I just feel better about myself*. *Now I actually cleaned up and organized my entire house last year" (C7)*.

Another client stated:

*"And yes*, *I just dare to show myself more in society" (C9)*.

HCPs confirmed the positive effects on the clients’ mental wellbeing:

*"And then you also see the effects of that*, *that they feel much better about themselves. And as a result, they’re stimulated to continue working on a healthy lifestyle" (HCP7)*.

### Theme 4: Preconditions for clients in changing health behaviour

#### Clients needed time and intensive guidance for behaviour change

Most clients were capable of following the extensive one-year intervention and participating in the SMILE group sessions but some needed more support than others. For example, the change from weekly to monthly meetings after six months was mentioned as a noticeable reduction of support and structure, which was experienced as undesirable by some clients. After the one-year intervention period ended, some clients asked about the possibility of continuing the intervention with booster sessions because they needed additional support.

*"Yes, well, I think just getting together once in a while for the finishing touches*. *That you just go through the important things again. You have the binder for guidance, but in daily life you are distracted by a lot of things, so you can lose focus a bit. Just to discuss things again and sit down together again and get that focus back" (C3)*.

HCPs stressed the importance of continued support over time for people with SMI to preserve and possibly further increase lifestyle changes. They recommended more guidance and additional individual face-to-face support for some clients during the SMILE intervention and the possibility of continuing group sessions after one year.

*"It really is a long process*. *And especially for this group of patients. Most important is to stick with it for a long time, to continue to support them" (HCP12)*.

#### Learning in practice was most beneficial

The practical activities in the SMILE intervention were regarded as most useful by clients. Sessions with a very practical approach were mentioned as the most appealing. These sessions contributed most towards improved awareness and actual lifestyle changes, as opposed to more information-driven sessions. These practical approaches were supported with visual aids, also appreciated by clients. One said:

*"We also received cards with foods, we had to put them in order from containing the most fats or sugar to the least fats or sugar*. *And that shocked people, how much sugar ice cream or an almond round contains" (C11)*.

In addition, another client mentioned that practical activities were preferred because otherwise it was difficult to concentrate for a longer period of time:

*"Yes, they were very good at that*. *And yes, we could not always concentrate for a long time, you cannot listen for an hour, you know, because then the concentration is gone and so this practical approach is just very nice" (C13)*.

HCPs confirmed the preference of clients for practical approaches:

*"People are very often visually oriented such as with those cards*, *then people become more enthusiastic" (HCP11)*.

## Discussion

The results of this study shed light on different aspects considered important when delivering a lifestyle intervention to people with SMI. These clients’ experiences and perceptions were complemented with the perspectives of their HCPs. The findings have led to suggestions for tailoring lifestyle interventions in the best possible way for individuals with SMI. Four overarching themes emerged from our analysis. We found that clients valued the SMILE intervention and experienced benefits from this intervention in a group-based setting. These benefits were not limited to weight loss or behaviour changes, but extended to the appreciation of peer support, improved awareness of health issues, improvements in psychosocial functioning and mental wellbeing. It was striking that for most clients, weight loss, which was the primary outcome in the RCT we conducted, was not the most important outcome; they felt positive about all improvements in the above-mentioned areas. For many clients, changing behaviour was complex and difficult to sustain, to a large extent due to the consequences of the mental illness, for example (residual) psychotic symptoms, depressive symptoms or impairments in cognitive functioning (in particular impaired concentration). It should be noted that achieving sustainable lifestyle changes and effects is difficult for almost all people who try to realize this. However, clients with SMI are confronted with a number of additional challenges, not only the previously mentioned consequences of the mental illness, but also the confrontation with stigma and self-stigma, reduced self-esteem, less social support, fewer financial resources, and reduced access to regular sports facilities [13, 23–25]. Additional support and a practical approach are needed to help clients find solutions for the challenges they encounter.

An interesting result was the great appreciation of both clients and HCPs for the group setting of the intervention. Before starting the study, many HCPs and management staff were sceptical about its group-based design. They predicted that people with SMI would not be interested in following a group-based lifestyle intervention for one year, due to lack of motivation and inability to function in a group. According to them, low adherence to the group sessions could jeopardize the success and effectiveness of the intervention programme. While we recognize that a group setting will not be ideal for all people with SMI, this turned out to be one of the most successful aspects of the SMILE intervention. Published research also suggests that people with SMI who follow a group-based lifestyle intervention experience the peer support essential for lifestyle behaviour change, in which being together with people who are ‘in the same boat’ is considered a key feature [26]. Therefore, we would advise always to consider group-based settings when delivering lifestyle interventions for people with SMI, though with constant vigilance for possible signs of drop-out of clients who are not capable of effectively participating in these. Such clients may benefit better from an individual approach. However, when tailoring the activities in group-based lifestyle interventions to the specific competencies, needs and preferences of clients, this individual approach may also be suitable, on condition that the group size is limited. In our opinion, people who could benefit most from a group-based intervention are those who are looking for (new) social contacts, enjoy social activities or are interested in learning from peers. Group sizes from seven to ten clients turned out to be workable in the delivery of the SMILE intervention.

Many clients in this study had cognitive impairments which may be important barriers to successful behaviour change for people with SMI. Cognitive impairments were also common in the STRIDE study [11], where participants often mentioned these as a barrier to effective participation in the intervention programme and, in the end, to behaviour change. In our study, HCPs mentioned the need for using practical approaches, setting small goals and delivering additional individual support to better meet the needs, strengths and limitations of the clients. In the STRIDE study [11], participants also cited depressive symptoms as having a significant negative influence throughout the intervention. Depressed mood influenced their eating behaviour, in many cases leading to overeating. In our study, clients also mentioned this and emphasized the importance of learning how to cope with emotional eating episodes. There is a known association between depression, emotional eating and obesity [27] and we therefore recommend more focus on emotional eating, preferably with the help of psychological interventions.

Notably, clients mentioned a variety of successful lifestyle changes throughout the intervention. For clients, even ‘small’ changes were highly important and perceived as great successes. As noted earlier, for many patients, weight loss was not the most important outcome. HCPs should be aware of the possible differences between their expectations and clients’ goals when it comes to weight loss versus other forms of lifestyle change. If weight loss is not the primary goal for all clients, it cannot be the primary criterion of success.

Other beneficial effects of the intervention mentioned by clients included improved daily rhythm and mental wellbeing. Focusing on a variety of healthy behaviours such as those concerning eating, physical activity, stress, sleep, and social participation, and how to integrate these in daily life can therefore be of great interest for many people with SMI.

Overall, the interest of clients in the lifestyle intervention was greater than expected. During the recruitment process, HCPs noted that clients were interested in joining the SMILE intervention, including some who did not meet our inclusion criterion of overweight. The assumption of some HCPs that clients have limited or no interest in lifestyle improvement does not seem to be justified. Therefore, possible participation in lifestyle interventions should always be discussed with clients.

### Strengths

We performed a process evaluation as part of a pragmatic RCT [16]. Our current study and the process evaluation were performed alongside each other, drawing on the same data [16]. Together they provide a comprehensive insight into process of implementation, related barriers to and facilitators of effective implementation, and the experiences of clients and HCPs with the lifestyle intervention. We added valuable scientific information to the quantitative analyses of the cost-effectiveness trial. In this way, we surpassed the ‘black box limitation’ of the RCT and provided explanatory data for the results of the trial.

### Limitations

Our results shed light on the experiences and perceptions of people with SMI who decided to enter the intervention but it does not say anything on how to encourage the engagement of those not interested in adopting healthy behaviours or not willing to participate in a structured, group-based lifestyle programme. Therefore, it is plausible that selection bias has occurred as people not interested in following a lifestyle intervention were not included in the study. It is important to view our findings with this in mind. A potential limitation might be the fact that only 48% of the client interviewees responded to the member check and agreed with the summary. However, we were satisfied with this response rate of 48%, because we didn’t expect a high response rate in this population of clients with SMI.

## Conclusions and implications for practice

The results of this qualitative study shed light on aspects that need to be considered when delivering a lifestyle intervention to people with SMI. To better accommodate people with SMI, we recommend incorporating the following aspects when delivering a lifestyle intervention:

- Integrate group-based elements in lifestyle interventions, but recognize that additional individual attention may be needed for some clients;
- Be aware of the time investment that will be needed for people with SMI to achieve stable lifestyle changes;
- Recognize the complexity of changing lifestyle behaviour for people with SMI and use practical and easy-to-understand activities during the intervention, e.g. supporting the intervention with visual aids and practical activities;
- Broaden the set of outcomes of lifestyle interventions beyond solely weight loss and metabolic parameters to include, for example, improved mental wellbeing, quality of life and social participation.

## Supporting information

S1 File (DOCX)
