# Supplementary material for: Experiences and perceptions of people with a severe mental illness and health care professionals of a one-year group-based lifestyle programme (SMILE)
Source: PLoS One. 2022 Aug 4;17(8):e0271990. doi: 10.1371/journal.pone.0271990 (PMC9352038; doi:10.1371/journal.pone.0271990)
Supplement: S1 File — (DOCX) [file pone.0271990.s001.docx]

**S1 Appendix Overview of SMILE intervention content**

| **Weekly sessions**  **(Initial phase)** | **Content of session** |
| --- | --- |
| Session 1 | Welcome and introduction to SMILE |
| Session 2 | Portion sizes |
| Session 3 | Energy balance and goal setting |
| Session 4 | Breakfast, physical activity and regular eating |
| Session 5 | Working on a healthy eating pattern |
| Session 6 | Eating healthy on a budget |
| Session 7 | Progress check |
| Session 8 | Meal planning |
| Session 9 | Your environment and social support |
| Session 10 | Adverse effects of medication and weight gain |
| Session 11 | Sleeping behaviour and coping with stress |
| Session 12 | Progress check and tackling negative thinking |
| Session 13 | Eating consciously |
| Session 14 | Over-eating and emotional eating |
| Session 15 | Eating out |
| Session 16 | Importance of physical activity |
| Session 17 | Meal planning and portion sizes |
| Session 18 | Progress check and problem solving |
| Session 19 | Social support |
| Session 20 | Pitfalls |
| Session 21 | Stagnation of progression |
| Session 22 | How to maintain weight loss |
| Session 23 | Coping with changes in mental health status |
| Session 24 | Celebrating successes |
| **Monthly sessions (Maintenance phase)** |  |
| Session 25-30 | Recap of former topics and free input |
|  | |

# S2 Appendix Topic lists

*Topic list for interviews with clients with SMI*

| **General** |
| --- |
| What did you think of the SMILE programme? |
| **Participation, suitability, what does the group know** |
| Why have you decided to participate in this programme?  What did you expect from the programme?  What did you find interesting that made you return to the sessions?  You did not come anymore, how did that come about? |
| **Effectiveness** |
| You have lost XX kilos after six months. How did that go?  What are you doing differently now?  Since participating, how are you feeling?  How did people react to SMILE at home? Did you experience support from your environment?  How was that for you? |
| **Adoption of the SMILE intervention** |
| In the programme, you have had guidance from [XX] and [XX], How did they do it? |
| **Group sessions and the group** |
| You have set goals, how did that go?  What was it like for you to set goals?  There was also a small work-out together with the group, what did you think about that?  Various topics have been discussed. What topics do you remember most?  How did you feel in the group? How did that come about?  If it were up to you, what would the group look like to you? |
| **The group** |
| How did you feel in the group? How did that come about?  If it were up to you, what would the group look like to you? |
| **Maintenance** |
| What would help you stay on the path where you are now? |
| **Final question:** Is there anything else you want to talk about? |

*Topic list for interviews with Healthcare professionals*

| **General** |
| --- |
| How did you like to give SMILE? |
| **Participation, suitability, what does the group know** |
| Do you think the intervention is in line with the SMI target group? |
| **Effectiveness** |
| What has changed in behavior during the SMILE programme?  Has the SMILE programme helped people change their behavior?  How is it that SMILE works for some people and not others?  What does it take to make SMILE successful with those it hasn't helped?  When is the intervention effective?  What are the main successful elements / components of SMILE?  What influence did the social environment / home situation have on the participants? |
| **Group sessions and the group** |
| Which group sessions have you remembered the most?  What was the best element and what was not?  How did the group interact with each other? What was the group dynamics like?  How did you deal with the group dynamics? |
| **Maintenance** |
| How can SMILE be improved?  How would you ideally view lifestyle programmes in people with SMI?  What advantages and disadvantages did the programme have? |
| **Final question**: Have we forgotten things you want to talk about? |
