# Supplementary material for: Experiences and perceptions of people with a severe mental illness and health care professionals of a one-year group-based lifestyle programme (SMILE)
Source: PLoS One. 2022 Aug 4;17(8):e0271990. doi: 10.1371/journal.pone.0271990 (PMC9352038; doi:10.1371/journal.pone.0271990)
Supplement: Attachment — Submitted filename: 20220526 Response to Reviewers.docx [file pone.0271990.s002.docx]

**Review Comments to the Author**

**Reviewer #1:**
Overall, I thought the research was well done and meaningful. I found the Introduction and Methods to be the weaker sections in that key aspects of the research were not always described clearly. The recommended revisions are relatively minor (that is, they should be easily achieved); however, they will substantively improve the quality of the manuscript.

**RESPONSE:** *We thank the reviewer for these positive comments and for the feedback on our manuscript. Please note that all lines mentioned in the responses in this document are based on the 'Revised Manuscript with Track Changes' document* when ‘’all markup’’ under display tracking is enabled.

**Concerns:**
**1.** Focus of the intervention: It would be helpful to acknowledge the primary focus of the lifestyle intervention (i.e., weight loss, physical activity) in the Abstract (Objective; lines 23-26) and Introduction. This was not clear until S1 Appendix was examined. Although there were hints of the focus (e.g., line 38, “Irrespective of weight loss …; description of the inclusion criteria (i.e., line 122-23), for the most part, the description of the program within the body of the manuscript was quite vague. This is problematic in that the descriptor “lifestyle intervention” is vague. Such programs can be narrow or broad. In the case of more narrow foci, programs can vary tremendously. It is recommended that the final paragraph of the introduction (lines 80-93) be revised to include a brief description of the program’s main focus.

**RESPONSE:** *Thank you for your comment. We agree that the current description is vague at certain places in the manuscript.*
**CHANGE:** *We added more information regarding the focus of the lifestyle intervention: in the abstract (lines 25-26), the introduction (lines 80-82) and the methods section (lines 110-113).*

**2.** The described research is part of a process evaluation of the intervention that was conducted in parallel with a pragmatic cluster-randomized control trial of the SMILE lifestyle intervention. While the main study was cited, it is not clear if the process evaluation of the intervention has been published. Moreover, it was not clear why the authors decided to present the qualitative data in a separate manuscript. While this decision may well be appropriate, it would be helpful if additional information was included about the primary process evaluation in the Study Design section of the Methods (lines 96-105). For example, was this evaluation quantitative in nature? If it has been published, a reference for that article should be provided (see lines 84 and 86). In revising this section, please ensure that the various components of the study as a whole are clearly described. Also, it may be pertinent, in the Discussion, for the authors to address the inter-relationships between the results of the two parts of the process evaluation. Are the two sets of results complementary? Are there any discrepancies?

**RESPONSE:** *The process evaluation was also qualitative in nature, however with a specific focus on the implementation process of the intervention, including a description of barriers and facilitators for effective implementation. As the present study and the process evaluation each have a different but complementary focus, and because it was impossible to summarize the wealth of information in one manuscript, we have prepared two different manuscripts.*
**CHANGE:** *The process evaluation of the study has recently been published, therefore we have added the reference of this publication when the process evaluation is mentioned in the manuscript. In addition, we explained the aim of the process evaluation (see also comment 3 from reviewer 2) in the introduction section (lines 84-86), in order to understand how the present study on the experiences of patients and health-care providers is related to the process evaluation. Finally, in the discussion (lines 459-462) we described the complementary relation of the process evaluation and the current article.*

**3.** SMILE Intervention (lines 107-123): Please provide more information about the structure of the program. How long was each session? What was the size of the groups (i.e., clients and HCPs; this information was finally provided in the Discussion section (lines 422-423))? Describe the SMI inclusion criterion. Were individuals with co-morbid mental health disorders eligible to participate? While a list of topics covered in the program is available in S1 Appendix, it would be helpful to describe briefly the kinds of topics addressed in the body of the manuscript.

**RESPONSE:** *For the inclusion of participants there was no distinction made between (co-morbid) mental health disorders in clients. All clients from participating FACT-teams were eligible to participate.*
**CHANGE:** *We added additional information regarding the SMILE intervention (lines 108 - 122), such as the timing of the sessions, group sizes, intervention topics and how many HCP’s were involved per session.*

**4.** Use of RE-AIM framework: It was stated that the interview guides were based on the RE-AIM framework (Glasgow et al., 2019). While this framework is very pertinent, it was not clear how it was used, even after reviewing the two interview guides (S2 Appendix). First, it may be helpful to describe more fully the nature of this framework and how it was used. That is, describe the fit between the framework and the interview guides. Presumably, RE-AIM was also considered when deciding who the participants would be in the current study. Some of the dimensions (e.g., implementation and maintenance; perhaps, adoption) are also pertinent to the implications of the research. Accordingly, it may be helpful to consider this framework in the Discussion section.

**RESPONSE:** *For the process evaluation and the current study on experiences of patients and professionals, the qualitative results were derived from the same data source (semi-structured interviews). Both articles are complementary to each other. We based the topics and interview guide on the RE-AIM framework which was explicitly used for the process evaluation to describe the process of implementation, including barriers and facilitators. In the analysis and writing of the current manuscript, the RE-AIM framework was no longer explicitly used, given its focus on experiences of clients and professionals with the lifestyle intervention.* *Therefore, we have not provided an extensive explanation of the RE-AIM framework and have not used it for data analysis and reporting for the current manuscript.*
**CHANGE:** *We have made some adjustments in the section Procedures to clarify the use of the RE-AIM framework in the two studies: the process evaluation and the current study on the experiences of clients and professionals.*

*The following sentences were added:*

- *The topics were based on the RE-AIM framework as this study was performed alongside the process evaluation which focused on the process of implementation of the intervention (Lines 143-145).*
- *These RE-AIM dimensions were incorporated throughout the interviews but were not explicitly used for the analysis of the data in the present study, given our focus on the experiences of clients and professionals (Lines 150-152)*.

**5.** Participants (Results): Please include a brief description of the kinds of SMI experienced by the participants. Please note that the tables are incorrectly referred to on lines 183-184. That is, Table IA concerns the HCPs while Table 1B is client-focused.

**RESPONSE:** *We thank the reviewer for this comment. The global description of the psychiatric diagnoses can be found in Table 1B. We purposely did not further specify these diagnoses in order to ensure privacy of the clients, as HCPs know which of their clients was interviewed for the study.*
**CHANGE:** *We corrected the references for Tables 1A and 1B.*

It was also noted in the Methods that during the second half of the intervention, individual phone support was available as an adjunct to the monthly group sessions (line 109-110). It is recommended that information about the frequency of these contacts be described in this section or in Table IB. Was this type of support addressed by clients in the interviews? This is especially important given that HCPS recommended offering increased individual face-to-face support for some clients (see lines 348-249; see also lines 418-419). Again, was this recommendation fairly general or are there sub-groups of clients that would benefit from this support?

**RESPONSE:** *This is an interesting question. We agree that better insight into the telephone contacts could provide valuable information. Unfortunately, we were not able to collect reliable data on the use of those contacts, despite our efforts in this regard. In the interviews, the telephone contacts were not explicitly put forward as being valuable or not. Therefore, we cannot provide more information about this.*

**6.** Results (Themes 1 and 2): The description of these themes seem to focus upon the experiences and perceptions of the majority of clients. The experiences of the minority, however, can be very pertinent – especially in considering the effectiveness of a program and steps to enhance the program. Accordingly, it may be helpful to describe some of the dissenting views. For example, it was stated that some participants wanted to gain more knowledge about lifestyle related issues (lines 206-207). What were the primary interests of the other clients? Did the interests seem to vary as a function of participant characteristics (e.g., gender, age, etc.)? The answers have help to refine and extend some of the recommendations made in the Discussion.

**RESPONSE:** *We agree that when addressing ‘the majority’ on some views it seems we leave out views of the minority. We agree that this seems to make the information in the results section incomplete, as views from minorities of the participants are also of interest. When looking into our data again and when considering how we elaborated themes 1 and 2, we may conclude that no specific views of a minority of patients emerged from the data. The way we phrased some of the perspectives of respondents with respect to themes 1 and 2 were complete, given the available data. The fact that with respect to some (sub)themes a majority of respondents articulated their perspectives explicitly, this often means that other respondents did not verbalize their perspective in an explicit way. This is inherent to the qualitative method followed, using a semi-structured interview protocol and an iterative process of data-collection and data-analysis (see also our response to Reviewer 2).*

*That said, we were able to make some adjustments. We modified some sentences to clarify the above-mentioned aspects. Further, we added additional reasons participants mentioned to join the sessions, in addition to the most common reasons we already mentioned under theme 1. In the end, no clear dissenting views emerged from our data to report in the results section. Also, the analysis showed no clear relationship between participant characteristics and articulated perspectives for both theme 1 and 2.* **CHANGE:** *We changed some phrasing of themes 1 and 2 in line with the above-mentioned clarification. In addition, we added some extra views for theme 1 regarding primary interests for joining the sessions (lines 208-210).*

**7.** Discussion (appropriateness of a group-based intervention): On line 408, the authors state that “a group setting will not be ideal for all people with SMI …” Any thoughts as to who will benefit most from a group-based intervention?

**RESPONSE:** *People who could benefit most from a group-based intervention are those who are looking for (new) social contacts, enjoy social activities or want to learn from peers.*
**CHANGE:** *We added the following sentence to the discussion section: In our opinion, people who could benefit most from a group-based intervention are those who are looking for (new) social contacts, enjoy social activities or are interested in learning from peers. (Lines 420-422).*

**8.** Discussion (cognitive impairments): On line 395, it was acknowledged that many of the clients had cognitive impairments and that these impairments may be a barrier for successful change. What kinds of cognitive impairments? The nature of these impairments may lead to specific recommendations/procedures for addressing this potential barrier.

**RESPONSE:** *We agree that it is important to specify the clients’ cognitive impairments in order to better apply procedures or recommendations to address this potential barrier. The data revealed that impairments in concentration were particularly mentioned.*
**CHANGE:** *We added this information in line 284(results) and line 395 (discussion).*

Comments related to the other specific Review Questions:
***2.** Has the statistical analysis been performed appropriately and rigorously?
The described study uses qualitative methods and, as such, the issue of statistical analysis per se is not relevant. Having said that, it is important to consider the thematic analysis methods used. Overall, the methods used to analyze the transcripts and identify emerging themes were appropriate. In addition, member checking was conducted (lines 158-160) to help demonstrate credibility of the data and identified themes. Importantly, they had a strong response rate.
It was indicated that two members of the study team reviewed and coded each transcript (line 163). However, no information was provided about the consistency (or congruence) of the two sets of codes and how differences were resolved. It is recommended that a brief description of this process be included.

**CHANGE:** *We added additional information regarding this process in line 169-172.*

The statement on Line 166 regarding the validity of the themes was unclear. It is unusual to talk about the validity of qualitative data or themes, more generally.

**RESPONSE:** *We agree this is unusual. Instead of valid/validity we now used the words ‘credible/credibility’ as these terms are more common in qualitative research.*

**CHANGE:** *We adapted this sentence to: (4) reviewing whether themes credibly represent the data (lines 166-167).*

***4.** Is the manuscript presented in an intelligible fashion and written in standard English?
Overall, the manuscript was clearly written in standard English. There were sections that were awkward such that it was hard to understand some of the points being made. Careful proof-reading and editing will be sufficient to address this comment.

**CHANGE:** *We have asked an English native speaker with experience with academic writing to edit the manuscript to make the article more clearly written in English. Several changes have been made throughout the manuscript.*

**Reviewer #2:**

While I have expertise in severe mental illness and knowledge of qualitative methods, I do not use such methods in my work. Rather, I appreciate the approach in terms of its yield of underlying processes that are invisible in RCTs and quantitative approaches. This study did not disappoint as I found it very useful and informative. Note that I cannot judge the statistics other than recognizing that those described are used in qualitative studies. The lack of fully publically available data does not surprise me given confidentiality matters for clinical samples. The authors make it available under appropriate conditions, in my view.

**RESPONSE:** *We thank the reviewer for these positive comments and for the feedback on our manuscript Please note that all lines mentioned in the responses in this document are based on the 'Revised Manuscript with Track Changes' document* when ‘’all markup’’ under display tracking is enabled*.*

In terms of suggestions to the authors, here is my list:
**1)** Abstract should be clear about the objectives so persons unfamiliar with SMILE will know what the study is about.

**RESPONSE:** *We agree this was unclear, therefore we adapted the aim description in the abstract.*
**CHANGE:** *We changed the objectives to make it more clear for people unfamiliar with SMILE (Lines 23-26).*

**2)** Extensive use of acronyms that are unexplained is problematic for clarity, e.g., what is FACT?

**CHANGE:** *We added the explanations for the acronyms FACT and SMILE to the manuscript.*

*FACT: Flexible Assertive Community Treatment*

*SMILE: Severe Mental Illness Lifestyle Evaluation*

**3)** Explain more of the technical jargon, e.g., what is "process evaluation" exactly? Look for jargon and clarify meaning so the work is consumable by persons not steeped in qualitative methods.

**RESPONSE:** *Thank you for your comment, we agree it is important to explain more technical jargon in the manuscript to make the work more accessible for people who are not steeped in qualitative methods.*
**CHANGE:** *We added explanations of the following technical jargon to the manuscript:*

*(1): Process evaluation (Lines 84-86, 101-102 and 458-463)*

*(2): Purposive sampling (Lines 129-137)*

*(3): Data saturation: (Lines 156-157)*

**4)** The authors state that selection bias possibly occurred. Of course it occured, be clear that it did.

**RESPONSE:** *We agree and changed this sentence in the discussion section.* **CHANGE:** *We changed the sentence to: Therefore, it is plausible that selection bias has occurred. (line 471).*

**5)** When describing themes, terms like most participants and some participants are frequent. I would suggest giving percentage to make things more concrete.

**RESPONSE:** *Giving percentages or numbers is uncommon in qualitative research. As an iterative process is used, topics were adapted throughout the interview period, and new topics were added in subsequent interviews, based on information collected in previous interviews. It is possible that some (new) topics were not discussed in previous interviews as these topics were not addressed in those interviews but were in later interviews. In this qualitative research the exact quantity is not of primary concern, but we do give an indication to give an impression of bigger or smaller importance.*
**CHANGE:** *We believe it is opportune to provide exact quantitative figures in terms of percentages as this is uncommon for qualitative studies.*

**6)** Address as a potential limitation that some participants attended a very small percentage of the 30 available sessions. What might be the impact or such.

**RESPONSE:** *We purposely invited clients with a low attendance rate in the intervention to participate in the interviews to learn about their perspectives as well (this was done as part of the purposive sampling strategy, of which we have now added an explanation on lines 129-137). Learning the perspectives of participants with a low attendance is of interest as only interviewing people with a high attendance (who we assumed would be more satisfied with the intervention) will give one-sided views. Therefore, we do not believe this is a limitation of the study.*
**CHANGE:** *We believe it is not necessary to address this as a potential limitation as these participants were purposively added to the sample, as part of our purposive sampling strategy.*

**7)** Likewise, as a limitation concern, what are the potential implications that only 48% of the client interviewees "responded to the member check and agreed with the summary". Why were they unresponsive at such a higher rate and does it degrade the contribution?

**RESPONSE:** *Beforehand, for this population of clients with severe mental illness we did not expect a high response for the member checks. We were satisfied with the response rate of 48%. All member checks we received from clients mentioned that no changes were needed to the provided summary. However we do agree this might be a potential limitation.*
**CHANGE:** *We have added this as a limitation in the limitations section in the discussion (lines 474-476).*
